# DHAV-1 2A1 Peptide – A Newly Discovered Co-expression Tool That Mediates the Ribosomal “Skipping” Function

**DOI:** 10.3389/fmicb.2018.02727

**Published:** 2018-11-15

**Authors:** Xiaoyao Yang, Qiurui Zeng, Mingshu Wang, Anchun Cheng, Kangcheng Pan, Dekang Zhu, Mafeng Liu, Renyong Jia, Qiao Yang, Ying Wu, Shun Chen, Xinxin Zhao, Shaqiu Zhang, Yunya Liu, Yanling Yu, Ling Zhang

**Affiliations:** ^1^Institute of Preventive Veterinary Medicine, Sichuan Agricultural University, Wenjiang, Chengdu, China; ^2^Key Laboratory of Animal Disease and Human Health of Sichuan Province, Sichuan Agricultural University, Chengdu, China; ^3^Avian Disease Research Center, College of Veterinary Medicine, Sichuan Agricultural University, Chengdu, China; ^4^School of Medicine, Shanghai Jiao Tong University, Shanghai, China

**Keywords:** DHAV-1, 2A1, ribosomal, “skipping”, motif, apply

## Abstract

Duck hepatitis A virus 1 (DHAV-1) belongs to the genus *Avihepatovirus* in the family *Picornaviridae*. Little research has been carried out on the non-structural proteins of this virus. This study reports that 2A1 protein, the first non-structural protein on the DHAV-1 genome, has a ribosomal “skipping” function mediated by a “-GxExNPGP-” motif. In addition, we prove that when the sequence is extended 10aa to VP1 from the N-terminal of 2A1, the ribosome “skips” completely. However, as the N-terminus of 2A is shortened, the efficiency of ribosomal “skipping” reduces. When 2A1 is shortened to 10aa, it does not function. In addition, we demonstrate that N^18^, P^19^ G^20^, and P^21^ have vital roles in this function. We find that the expression of upstream and downstream proteins linked by 2A1 is different, and the expression of the upstream protein is much greater than that of the downstream protein. In addition, we demonstrate that it is the nature of 2A1 that is responsible for the expression imbalance. We also shows that the protein “cleavage” is not due to RNA “cleavage” or RNA transcription abnormalities, and the expressed protein level is independent of RNA transcriptional level. This study provides a systematic analysis of the activity of the DHAV-1 2A1 sequence and, therefore, adds to the “tool-box” that can be deployed for the co-expression applications. It provides a reference for how to apply 2A1 as a co-expression tool.

## Introduction

The family *Picornaviridae* comprises small non-enveloped viruses with RNA genomes of 6.7–10.1 kb, and contains more than 30 genera and 75 species (Zell et al., 2017). Picornaviruses are single-stranded positive-sense RNA viruses whose genome contains a single open reading frame that encodes a large polyprotein precursor, which consists of the structural protein P1 and the non-structural proteins P2 and P3. The polyproteins are then cleaved into mature, independent proteins by self-encoding proteases such as 2A and 3C and so on (Sun et al., 2016, 2017). The genome encodes a few mature proteins, approximately 11. It can be simply divided into VP4-VP2-VP3-VP1-2A-2B-2C-3A-3B-3C-3D (Wen et al., 2015; Zhang et al., 2017). The 2A protein is one of the non-structural proteins, and of all the proteins encoded by the genome it is the least conserved. Interestingly, several types of 2A have been reported based on their structures and corresponding functions (Yang et al., 2017). In addition, some viruses encode multiple 2A-like sequences – a picornavirus related to duck hepatitis A virus 1 (DHAV-1) was shown to have a total of six 2A proteins, including four ribosome “skipping” 2A proteins (Wang et al., 2014). The multiple 2A proteins present in one virus may execute the same or different functions, depending on their structure (Johansson et al., 2002; Boros et al., 2013; Barbknecht et al., 2014; Lange et al., 2014). Each type has different motifs and corresponding functions.

DHAV-1 belongs to the genus *Avihepatovirus* within the family *Picornaviridae* (Tseng et al., 2007). The disease it causes is an acute, highly contagious, often fatal infection of both young ducklings and adult ducks (Mao et al., 2017), causing substantial economic losses in the duck industry worldwide. Gross pathological changes appear chiefly in the liver, which is enlarged with petechial and larger hemorrhages (Ou et al., 2017a). Spleen enlargement and swelling of the kidneys may also be apparent (Ou et al., 2017b). Microscopic changes in the liver are characterized by extensive hepatocyte necrosis, together with varying degrees of inflammatory cell infiltration.

DHAV-1 has the classical picornaviral genome structure. However, it encodes three tandem 2A proteins named 2A1, 2A2 and 2A3 – each with a different function. Briefly, the 2A2 protein possesses a GTPase activity and mediates cell apoptosis (Cao et al., 2016) and the 2A3 protein is associated with the H-rev107 protein family that is involved in cell proliferation (Wei et al., 2015). 2A1 comprises two parts, an N-terminal region (without sequence conservation) and a conserved motif comprising the seven C-terminal residues of 2A1 and the N-terminal proline of the downstream 2A2 (-GxExNPG↓P-). The motif induces a co-translational “cleavage” event that releases P1-2A1 from the non-structural proteins P2 and P3. Termed “ribosome skipping,” “StopGo,” and “Stop-Carry On” translation (Donnelly et al., 2001a; Brown and Ryan, 2010). The ribosome skipping was first suggested as a mechanism by Donnelly et al. (2001b). This translational recoding event predicts three alternative outcomes, after release of the nascent polypeptide: (i) translation of the downstream sequences resumes; or (ii) ribosomes terminate translation; or occasionally (iii) no recoding occurs, the peptide bond is formed and the protein is synthesized in the normal manner. The length of the 2A used is important for cleavage *in vitro* and *in vivo* – higher cleavage efficiency was reported when longer versions of foot-and-mouth disease virus (FMDV) 2A, with N-terminal extensions derived from capsid protein 1D upstream of 2A, were used (Donnelly et al., 2001a; Luke et al., 2008; Minskaia et al., 2013). Furthermore, the cleavage efficiency of shorter 2As is improved by the insertion of various spacer sequences, such as –SGS or –GSG immediately upstream of 2A (Szymczak et al., 2004; Holst et al., 2006; Provost et al., 2007). In general, mutations of conserved amino acids have more pronounced effects than changes to non-conserved ones (Donnelly et al., 2001a; Sharma et al., 2012). Finally, gene order effects on the cleavage efficiency, stability and subcellular targeting need to be considered (Rothwell et al., 2010; Appleby et al., 2013).

Given the similarity of DHAV-1 2A1 to FMDV 2A, we wanted to know whether DHAV-1 2A1 also possesses the ribosomal “skipping” function. Through experiments, we demonstrated that “-GxExNPGP-” does mediate this function. Two proteins linked by 2A1 can be expressed as separate individual proteins. In addition, we proved that the ribosomal “skipping” is not complete in all cases, which means that the two proteins linked by 2A1 could be expressed as a single fusion protein. The upstream protein carries 2A1 at its C-terminus and the downstream protein carries an amino acid P at its N-terminus. The number of amino acids upstream of the motif affects the efficiency of the “skipping.” Additionally, the number of amino acids containing this motif must be greater than 10 to allow the activity to occur. We also performed an alanine scan mutation on the motif to explore which amino acids are essential. In addition, we proved that the undivided fusion protein does not divide over time. This illustrates that the activity occurs during the translation process. From the experimental results, we also found that the expression of the upstream protein is much higher than that of the downstream protein. We proved that the different levels of protein expression are not due to the reporter genes or the order of the genes, but to the nature of 2A1. We also shows that the protein “cleavage” is not due to RNA “cleavage” or RNA transcription abnormalities, and the expressed protein level is independent of RNA transcriptional level.

## Materials and Methods

### Construction of Plasmids

DEF (duck embryo fibroblast) cells were infected with the DHAV-1 virus, and the total RNA of the infected cells was extracted and reverse transcribed to cDNA. The 2A1 fragment was amplified from the cDNA. EGFP and RFP fragments were amplified from pEGFP-N1 and pDsRed-Express-C1 vectors, respectively (amplification primers are shown in Table 1). Then, the amplified EGFP, 2A1 and RFP fragments were overlapped to the fusion fragment EGFP-2A1-RFP by overlapping PCR technology, and the EGFP-RFP fusion fragment was obtained by overlapping the EGFP and RFP fragments. Then, pcDNA3.1(+) and the two fusion fragments were digested with *Bam*HI and *Xho*I, and the digested fragments were cloned into the digested pcDNA3.1(+) vector to produce the two target plasmids. As well as the pcDNA3.1-EGFP-2A1-RFP and pcDNA3.1-EGFP-RFP, we constructed five other plasmids with different lengths of 2A1 sequence (VP1 + 2A1 = 30aa/25aa, extending the N-terminus of 2A1 to VP1; 2A1 = 18aa/12aa/10aa, shortening the N-terminus of 2A1). 2A1 of different lengths was amplified by the same method to construct the different pcDNA3.1-EGFP-(VP1)-2A1-RFP plasmids. All primers are shown in Table 1. The EYFP fragment in pcDNA3.1-EYFP-2A1-RFP (2A1 = 20aa) was amplified from pEYFP-N1 using F1 and R1 (from the pcDNA3.1-EGFP-2A1-RFP: 2A1 = 20aa) (see Table 1), and the construction method of the plasmid was as above. In plasmid pcDNA3.1-RFP-2A1-EGFP (2A1 = 20aa), the amplification primers for the RFP fragment were as follows: forward primer, 5^′^-*CGCGGATCCCGCCACCATGGCCTCCTCCG*-3^′^; reverse primer, 5^′^-*TTGTTTCTAATTTGGTCAGACAGGAACAGGTGGTGGCGGC*-3^′^. The amplification primers for the 2A1 fragment were as follows: forward primer, 5^′^-*GCCGCCACCACCTGTTCCTGTCTGACCAAATTAGAAACAA*-3^′^; reverse primer, 5^′^-*TCCTCGCCCTTGCTCACCATAGGTCCTGGGTTCGGTTCCA*-3^′^. The amplification primers for the EGFP fragment were as follows: forward primer, 5^′^-*TGGAACCGAACCCAGGACCTATGGTGAGCAAGGGCGAGGA*-3^′^; reverse primer, 5^′^-*CCGCTCGAGTTACTTGTACAGCTCGTCCA*-3^′^.

**Table 1:** The primers used to amplify the EGFP, (VP1) + 2A1, and RFP fragment in pcDNA3.1-EGFP-(VP1)-2A1-RFP and pcDNA3.1-EGFP-RFP.

pcDNA3.1-EGFP-(VP1) + 2A1-RFP: (VP1) + 2A1 = 30aa	Amplify EGFP	*F1: CGCGGATCCCGCCACCATGGTGAGCAAGG*
		*R1: AGATCGAGTTCAAATGCTAGCTTGTACAGCTCGTCCATGC*
	Amplify (VP1) + 2A1 = 30aa	*F2: GCATGGACGAGCTGTACAAGCTAGCATTTGAACTCGATCT*
		*R2: ACGTCCTCGGAGGAGGCCATAGGTCCTGGGTTCGGTTCCA*
	Amplify RFP	*F3: TGGAACCGAACCCAGGACCTATGGCCTCCTCCGAGGACGT*
		*R3: CCGCTCGAGCTACAGGAACAGGTGGTGGCGGC*
pcDNA3.1-EGFP-(VP1) + 2A1-RFP: (VP1) + 2A1 = 25aa	Amplify EGFP	*F1: as above*
		*R1: TCAGATTCAATTTCCAGATCCTTGTACAGCTCGTCCATGC*
	Amplify (VP1) + 2A1 = 25aa	*F2: GCATGGACGAGCTGTACAAGGATCTGGAAATTGAATCTGA*
		*R2: as above*
	Amplify RFP	*F3: as above*
		*R3: as above*
pcDNA3.1-EGFP-2A1-RFP: 2A1 = 20aa	Amplify EGFP	*F1: as above*
		*R1: TTGTTTCTAATTTGGTCAGACTTGTACAGCTCGTCCATGC*
	Amplify (VP1) + 2A1 = 20aa	*F2: GCATGGACGAGCTGTACAAGTCTGACCAAATTAGAAACAA*
		*R2: as above*
	Amplify RFP	*F3: as above*
		*R3: as above*
pcDNA3.1-EGFP-2A1-RFP: 2A1 = 18aa	Amplify EGFP	*F1: as above*
		*R1: TCTTTCTTGTTTCTAATTTGCTTGTACAGCTCGTCCATGC*
	Amplify (VP1) + 2A1 = 18aa	*F2: GCATGGACGAGCTGTACAAGCAAATTAGAAACAAGAAAGA*
		*R2: as above*
	Amplify RFP	*F3: as above*
		*R3: as above*
pcDNA3.1-EGFP-2A1-RFP:2A1 = 12aa	Amplify EGFP	*F1: as above*
		*R1: ACTCCTTCAGTAGTGAGATCCTTGTACAGCTCGTCCATGC*
	Amplify (VP1) + 2A1 = 12aa	*F2: GCATGGACGAGCTGTACAAGGATCTCACTACTGAAGGAGT*
		*R2: as above*
	Amplify RFP	*F3: as above*
		*R3: as above*
pcDNA3.1-EGFP-2A1-RFP:2A1 = 10aa	Amplify EGFP	*F1: as above*
		*R1: GGTTCCACTCCTTCAGTAGTCTTGTACAGCTCGTCCATGC*
	Amplify (VP1) + 2A1 = 10aa	*F2:GCATGGACGAGCTGTACAAGACTACTGAAGGAGTGGAACC*
		*R2: as above*
	Amplify RFP	*F3: as above*
		*R3: as above*
pcDNA3.1-EGFP-RFP	Amplify EGFP	*F1: as above*
		*R1:GAGGAGGCCATGGTGGCGTTCTTGTACAGCTCGTCCATGC*
	Amplify RFP	*F2:GCATGGACGAGCTGTACAAGAACGCCACCATGGCCTCCTC*
		*R2: as above*

### Cell and Plasmid Transfection

The primary DEFs were made using a 9-day-old duck embryo in DMEM containing 10% fetal bovine serum. The DEFs were cultured in six-well culture cluster plates at 80% confluence. Transient transfection was performed using Lipofectamin^TM^ 3000 (Invitrogen) to introduce the plasmids into the DEFs. Cells were lysed in 200 μl cell lysis buffer (Beyotime) containing 1% PMSF at 48 h post-transfection. Cell lysate was centrifuged, and the supernatant was collected and subjected to western blot analyses.

### *In vitro* Transcription

For *in vitro* transcription, plasmids containing different length 2A1 sequences were linearized at the *Xho*I site downstream of the open reading frame. The linearized DNA templates were purified and used for a quick transcription reaction using a High Yield Capped RNA Transcription kit (Invitrogen). After transcription, the template DNA was removed using TURBO DNase. The RNA was purified by the lithium chloride precipitation method. RNA integrity was assessed by electrophoresis using an ethidium bromide-stained agarose gel (1%), in TBE buffer.

### Real-Time Fluorescent Quantitative Reverse Transcription PCR (qRT-PCR)

For quantification of the EGFP, “skipping” site and RFP, standard curves were generated by amplification of serial dilutions of known concentrations of purified DNA templates ranging from 7 × 10^9^ to 7 × 10^2^ copies/μl. The transfected cells in five replicate wells were lysed in 1 ml RNAiso plus (Takara) per well and the RNA recovered by phenol:chloroform extraction. The isolated RNA was transcribed with a PrimeScript^TM^ RT reagent kit with gDNA Eraser (Takara). qRT-PCR was performed on the transcribed cDNA with Green^TM^ TB Premix Ex Taq II (Takara).

### The Alanine Scanning Mutagenesis of the Key Amino Acids “-G^14^xE^16^xN^18^P^19^G^20^/P^21^-”

The point mutations were carried out on pcDNA3.1-EGFP-2A1-RFP (2A1 = 20aa). The six key amino acids were all mutated to alanine in turn using the Fast Mutagenesis System (Transgen). The N^18^ couldn’t be mutated to A at one time, so it was mutated to a synonymous amino acid N’ firstly. Then the N’ was mutated to A. The mutant primers were designed as Table 2.

**Table 2:** The primers used to mutate the critical amino acids.

G^14^	G-A	*F: TCACTACTGAAGCAGTGGAACCGAA*
		*R: TTCGGTTCCACTGCTTCAGTAGTGA*
E^16^	E-A	*F: CTGAAGGAGTGGCACCGAACCCAGG*
		*R: CCTGGGTTCGGTGCCACTCCTTCAG*
N^18^	N-N’	*F1:GGAGTGGAACCGGACCCAGGACCTA*
		*R1: TAGGTCCTGGGTCCGGTTCCACTCC*
N^18^	N’-A	*F2: GAGTGGAACCGGCCCCAGGACCTAT*
		*R2: ATAGGTCCTGGGGCCGGTTCCACTC*
P^19^	P-A	*F: GTGGAACCGAACGCAGGACCTATGG*
		*R: CCATAGGTCCTGCGTTCGGTTCCAC*
G^20^	G-A	*F: AACCGAACCCAGCACCTATGGCCTC*
		*R: GAGGCCATAGGTGCTGGGTTCGGTT*
P^21^	P-A	*F: CCGAACCCAGGAGCTATGGCCTCCT*
		*R: AGGAGGCCATAGCTCCTGGGTTCGG*

### Western Blotting Analysis

Samples were collected as described above and the protein concentration was determined. Samples were fractionated by SDS-PAGE electrophoresis and then transferred to PVDF membrane, which was blocked with 5% non-fat dry milk at room temperature for 1 h. The membranes were incubated overnight at 4°C with mouse anti-EGFP antibodies (Beyotime) mouse anti-RFP antibodies (TDYbio) or mouse anti-β antibodies (Abmaking). The membranes were washed three times with TBS-Tween and incubated for 1 h at room temperature with HRP-conjugated goat anti-mouse IgG (Beyotime). The membranes were then washed three times with TBS-Tween and bound proteins were detected using an ECL chromogenic kit (Takara). The intensities of the signals for the bound proteins were, when necessary, quantitated using ImageJ2x software.

### Calculation Method for the Comparison of Expression

The protein bands were quantified by Image J2x. According to the ribosomal “skipping” function, when using 2A1 to link protein A and protein B (which is indicated as A-2A1-B), if the ribosome “skips,” the expressed protein would be the independent A-2A1 and B proteins; if the ribosome does not “skip,” the expressed protein would be the fusion protein A-2A1-B. Therefore, the total amount of protein expression should be the amount of upstream protein A resulting from ribosomal “skipping” plus the amount of fusion protein A-2A1-B resulting from ribosomal “unskipping” (which is indicated as A-2A1 + A-2A1-B). The expression efficiency of upstream A protein should be A-2A1/(A-2A1 + A-2A1-B). The expression efficiency of downstream B protein should be B/(A-2A1 + A-2A1-B). GraphPad Prism7 was used for data statistics.

## Results

### Sequence Alignment of Aphthovirus-Like 2A

**FIGURE 1:**
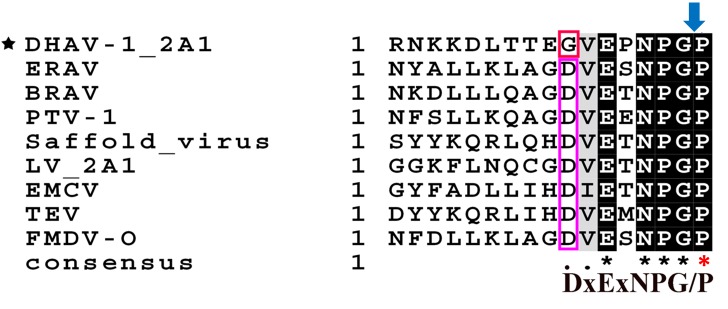
Sequence alignment of *Aphthovirus*-like 2As with DHAV-1 2A1. The amino acid P indicated by the red asterisk is the first amino acid of the protein following 2A1. Pink rectangle is a conservative amino acid D. The red rectangle is the only non-conserved amino acid G of the DHAV-1 2A1 peptide. The dark shaded part is a completely conserved part. The blue arrow represents the ribosomal “skipping” site.

Several common aphthovirus-like 2A sequences were selected to align with the DHAV-1 2A1 sequence (Figure 1). We can see that all of the 2A sequences are conserved except the DHAV-1 2A1 protein, which is only one amino acid different. We wondered whether this different DHAV-1 2A1 would also have a ribosomal “skipping” function.

### The Ribosomal “Skipping” Function of DHAV-1 2A1

**FIGURE 2:**
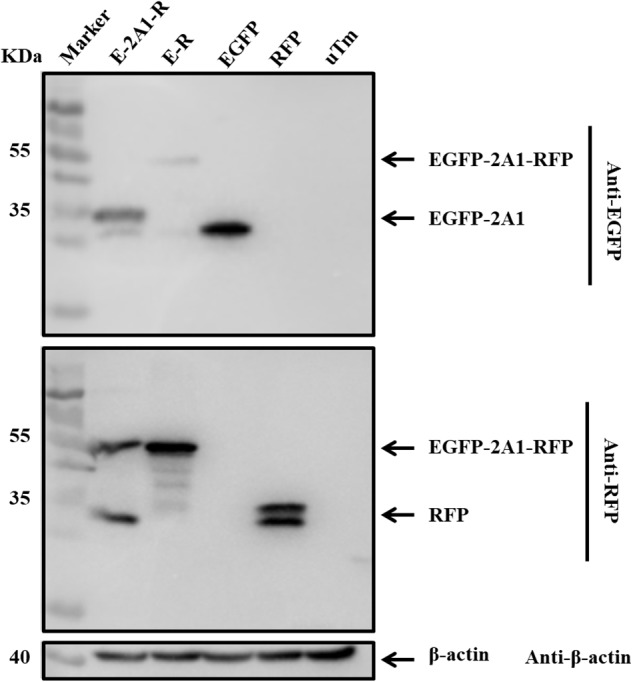
DHAV-1 2A1 mediates ribosomal “skipping” function. The expression of pcDNA3.1-E-2A1-R and control construct pcDNA3.1-E-R. The top half was immunoblotted with EGFP monoclonal antibodies (mAb). The bottom half was immunoblotted with RFP mAb. EGFP and RFP represent the control plasmid pEGFP-N1 and pDsRed-Express-C1, respectively. uTm represent the sample which was not transfected.

We linked two fluorescent reporter genes for EGFP and RFP with or without 2A1 to construct two vectors, EGFP-2A1-RFP and EGFP-RFP. To exclude the effects of redundant sequences, we used overlapping PCR techniques to get the EGFP-2A1-RFP and EGFP-RFP fragments. There are no other bases between each gene. The plasmids were used to transfect DEF cells in order to express the target proteins. Immunoblotting showed that the EGFP-2A1-RFP expressed proteins of small molecular weight, which were consistent with the size of GFP and RFP when immunoblotted with EGFP and RFP antibodies, respectively. However, the EGFP-RFP expressed a large molecular weight protein that conformed to the sum of the EGFP and RFP sizes (Figure 2). This experiment was indicative that the DHAV-1 2A1 mediates a ribosomal “skipping” function. The proteins linked by 2A1 were expressed as two individual proteins, with the 2A1 in the C-terminal of the upstream protein and the amino acid P in the N-terminal of the downstream protein.

### The Influence of N-Terminal Extension or Truncation in 2A1 on the Ribosomal “Skipping” Function

**FIGURE 3:**
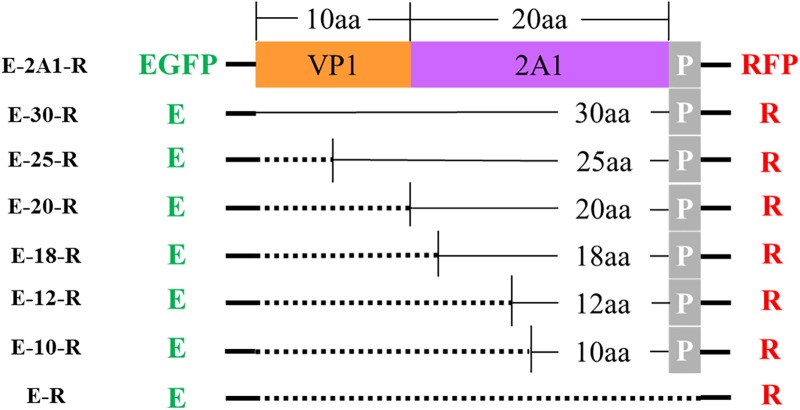
Schematic representation of various plasmids with different lengths. E: EGFP reporter gene; R: RFP reporter gene; P represents the first amino acid P of 2A2; the dotted line indicates that this fragment is absent.

**FIGURE 4:**
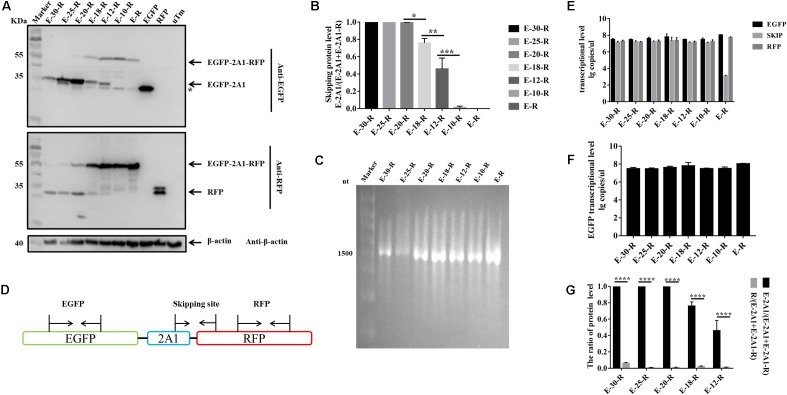
The influence of N-terminal extension or truncation in 2A1 on ribosomal “skipping” function. **(A)** Expression of various lengths of plasmids. The top half was immunoblotted with EGFP mAb. E-30-R represents the pcDNA3.1-EGFP-(VP1) + 2A1-RFP (VP1 + 2A1 = 30aa). E-25-R represents the pcDNA3.1-EGFP-(VP1) + 2A1-RFP (VP1 + 2A1 = 25aa). E-20-R represents the pcDNA3.1-EGFP-2A1-RFP (2A1 = 20aa). E-18-R represents the pcDNA3.1-EGFP-2A1-RFP (2A1 = 18aa). E-12-R represents the pcDNA3.1-EGFP-2A1-RFP (2A1 = 12aa). E-10-R represents the pcDNA3.1-EGFP-2A1-RFP (2A1 = 10aa). E-R represents the pcDNA3.1-EGFP-RFP. EGFP and RFP represent the control pEGFP-N1 and pDsRed-Express-C1, respectively. uTm represent the sample which was not transfected. The top half was immunoblotted with EGFP mAb. The bottom half was immunoblotted with RFP mAb. Asterisk refers unusual translational profiles. Two experimental materials were from the same batch of samples. **(B)** Protein expression level of EGFP reporter gene upstream different lengths of 2A1. **(C)** *In vitro* transcribed RNA agarose gel electrophoresis of each linear plasmids. **(D)** Schematic representation of the amplified fragments in qRT-PCR. **(E)** Transcriptional level of three fragments in each plasmids. **(F)** Transcriptional level of EGFP reporter gene upstream different lengths of 2A1. **(G)** Expression comparison of the upstream and downstream protein linked by 2A1. Five biological duplication were performed in each panels. Statistically significant differences were determined by one-way ANOVA. ^∗^*P* < 0.05, ^∗∗^*P* < 0.01, ^∗∗∗^*P* < 0.001, ^∗∗∗∗^*P* < 0.0001 indicate the level of statistical significance of differences between different groups.

From the experiments above, we found that the ribosomal “skipping” was not fully complete, resulting in the expression of some large fusion protein. To assess whether the sequences around the motif affect its function, we used different lengths of 2A1 to link the reporter genes and assessed the completion of the ribosomal “skipping.” We extended the N-terminus of 2A1 to the C-terminus of VP1 and shortened the N-terminus of 2A1 to different lengths (Figure 3). All the plasmids were well expressed (Figure 4). However, protein expression was different when using different lengths of linkers. When the amino acid number of the linker was 30, no large fusion protein was observed, and only the divided small proteins were detected. When the amino acid number of the linker was 25, a small amount of large protein appeared, but large amounts of small divided proteins were still detected (Figure 4A). With the reduction in the number of the N-terminal amino acids, the expression of the divided small proteins was gradually reduced. Correspondingly, the expression of large fusion protein was increased (Figure 4B). Moreover, no divided small proteins could be detected and only a large fusion protein was present when 2A1 had only 10aa. This was indicative that 2A1 could not perform ribosomal “skipping” when the amino acid number was 10 or less. These results supported the notion that the upstream amino acids of this motif also play an important role in the ribosomal “skipping” function. In addition, all translational profiles showed a smaller band under the EGFP-2A1 band compared to the blank control (indicated by an asterisk in the figure). We assume that an abnormal termination of translation may occur within the upstream protein, but this needs further confirmation.

In order to evaluate whether the translational products being separated or not was due to RNA abnormalities caused by transcription, we performed *in vitro* transcription of the plasmids with a eukaryotic *in vitro* transcription system. RNA electrophoresis showed that all plasmids were only transcribed to a single RNA with the size corresponding to the entire overlapping fragment (Figure 4C). Therefore, the difference in the proteins expressed is not caused by abnormal RNA transcription. To further confirm this finding, we used real-time fluorescent quantitative PCR to detect the RNA level of EGFP, “skipping” site and RFP fragment in all of the transfected samples (Figure 4D). We firstly established the respective standard curve of EGFP, “skipping” site and RFP to get the following equations: *Y* = -3.244*X* + 37.294 (EGFP), *Y* = -3.276*X* + 37.558 (“skipping” site) and *Y* = -3.315*X* + 37.345 (RFP) (*Y* is the threshold cycle; *X* is the log of the starting quantity) (Supplementary Figure 1). The three qRT-PCR detection ranges were all 10^4^∼10^8^. From the qRT-PCR results, it can be seen that except for E-R, the three RNAs in each group were at a high level and the levels were similar (Figure 4E). In addition, if the RNA were cleaved during transcription, the RNA at the “skipping” site would not be detected. This indirectly indicates that the RNA is not cleaved at the “skipping” site, and the protein cleavage to GFP and RFP is not derived from the transcriptional phase. In addition, the RNA level for GFP is similar between each group, indicative that the decrease of the EGFP protein level is also independent of the transcription level (Figure 4F).

### The Ribosomal “Skipping” Function Occurs During Translation and the Resulting Proteins Are Stable

**FIGURE 5:**
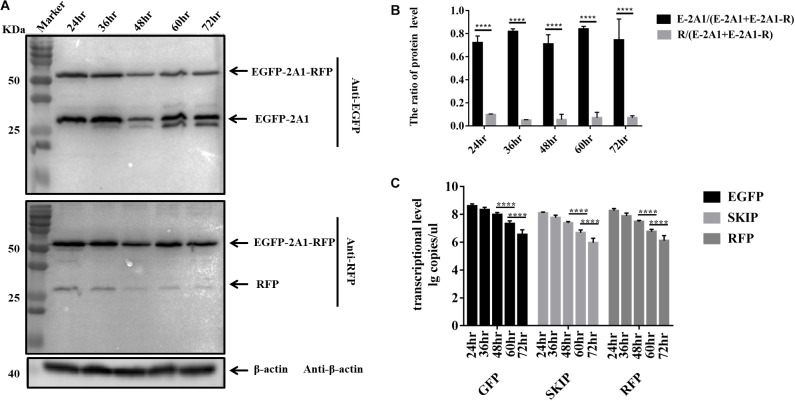
The ribosomal “skipping” function occurs during translation and undivided proteins do not separate over time. **(A)** The expression of pcDNA3.1-E-12-R at different points in time. The top half was immunoblotted with GFP mAb. The bottom half was immunoblotted with RFP mAb. Two experimental materials were from the same batch of samples. **(B)** Expression comparison of the upstream and downstream protein at different points in time. **(C)** Transcriptional level of three fragments in each points. Five biological duplication were performed in each panels. Statistically significant differences were determined by one-way ANOVA. ^∗^*P* < 0.05, ^∗∗^*P* < 0.01, ^∗∗∗^*P* < 0.001, ^∗∗∗∗^*P* < 0.0001 indicate the level of statistical significance of differences between different groups.

It has been reported that the ribosomal “skipping” function mediated by aphthovirus-like 2A occurs during translation. That is, the large fusion protein caused by “unskipping” cannot be divided into small proteins later on after translation is completed. We compared the protein expression of the pcDNA3.1-E-12-R plasmid at different time points. The pcDNA3.1-E-12-R plasmid was used to observe the large fusion proteins more clearly. The results were indicative that protein expression is identical from 24 to 72 h (Figures 5A,B). Moreover, early on in the first 24 h, it accumulated to its highest level. The resulting EGFP and RFP proteins also did not degrade over time. This indicated that they are stable in the cell. The RNA level at the corresponding time point showed that the RNA reached the highest level at 24 h, and it began to decrease at 48 h (Figure 5C). The transcription level did not affect the protein expression level. Therefore, this result supported the notion that the ribosomal “skipping” function is a translational mechanism.

### The Expression Imbalance Between the Upstream and Downstream Protein Linked by 2A1

**FIGURE 6:**
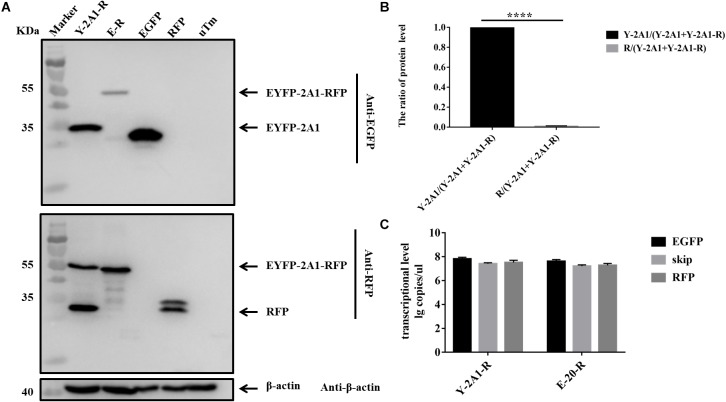
The expression level and RNA level of pcDNA3.1-EYFP-2A1-RFP. Experimental materials of each panel were from the same batch of samples. **(A)** The protein expression of pcDNA3.1-EYFP-2A1-RFP. The top half was immunoblotted with EGFP mAb. The bottom half was immunoblotted with RFP mAb. EGFP and RFP represent the control plasmid pEGFP-N1 and pDsRed-Express-C1, respectively. uTm represent the sample which was not transfected. **(B)** Expression comparison of upstream EYFP and downstream RFP in pcDNA3.1-EYFP-2A1-RFP**. (C)** The transcriptional level of pcDNA3.1-EYFP-2A1-RFP. Five biological duplication were performed in each panels. Statistically significant differences were determined by one-way ANOVA. ^∗^*P* < 0.05, ^∗∗^*P* < 0.01, ^∗∗∗^*P* < 0.001, ^∗∗∗∗^*P* < 0.0001 indicate the level of statistical significance of differences between different groups.

**FIGURE 7:**
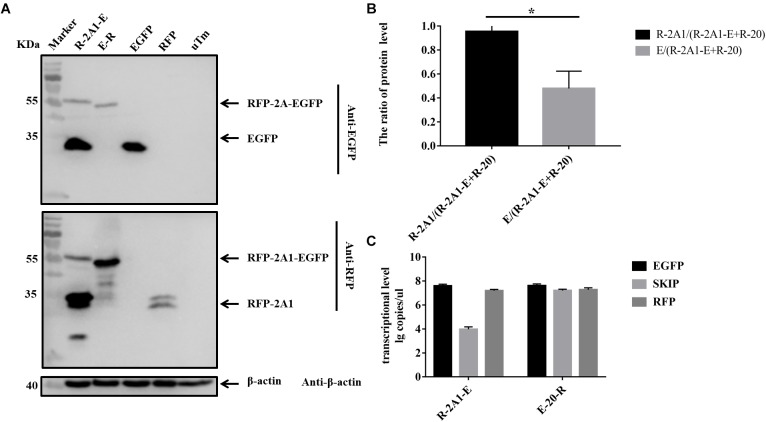
The expression level and RNA level of pcDNA3.1-RFP-2A1-EGFP. Experimental materials of each panel were from the same batch of samples. **(A)** The protein expression of pcDNA3.1-RFP-2A1-EGFP. The top half was immunoblotted with EGFP mAb. The bottom half was immunoblotted with RFP mAb. EGFP and RFP represent the control plasmid pEGFP-N1 and pDsRed-Express-C1, respectively. uTm represent the sample which was not transfected. **(B)** Expression comparison of upstream RFP and downstream EGFP in pcDNA3.1-RFP-2A1-EGFP**. (C)** The transcriptional level of pcDNA3.1-RFP-2A1-EGFP. Five biological duplication were performed in each panels. Statistically significant differences were determined by one-way ANOVA. ^∗^*P* < 0.05, ^∗∗^*P* < 0.01, ^∗∗∗^*P* < 0.001, ^∗∗∗∗^*P* < 0.0001 indicate the level of statistical significance of differences between different groups.

In the above experiments, we found that in western blots the EGFP band was always much brighter than the RFP band when detecting the same sample. Quantitative analysis revealed that the expression of EGFP was much greater than that of RFP (Figures 4G, 5B). This showed that the expression of upstream and downstream proteins linked by 2A1 was not identical. In some studies, it has been speculated that this is due to the nature of the reporter gene EGFP (Chan et al., 2011). However, our results do not support this view. We first replaced the upstream EGFP-encoding gene with a reporter gene for EYFP. The expression of the upstream protein was still higher than that of the downstream protein (Figures 6A,B). Therefore, we suspected that EGFP was not responsible for the inhibition of the expression of the downstream protein. Since EYFP is derived from EGFP, in order to further prove our conjecture, we reversed the order of upstream EGFP and downstream RFP. It was found that the expression of upstream RFP was higher than that of the downstream EGFP (Figures 7A,B). Then, we also investigated the transcription levels of the two samples, and we found that the transcription levels were high and similar in each of the samples (Figures 6C, 7C). Moreover, they all have similar RNA levels to E-20-R (the RNA level was not detected at the skipping site due to the order in which the upstream and downstream genes were exchanged in the RFP-2A1-EGFP). Therefore, the imbalance of protein expression was also independent of the level of transcription. From these experiments, we can conclude that it was not EGFP that caused the expression imbalance of the proteins linked by 2A1. We prefer the explanation that the unique nature of the 2A1 peptide is responsible for this phenomenon.

### The Influence of Critical Amino Acids on the Ribosomal “Skipping” Function

**FIGURE 8:**
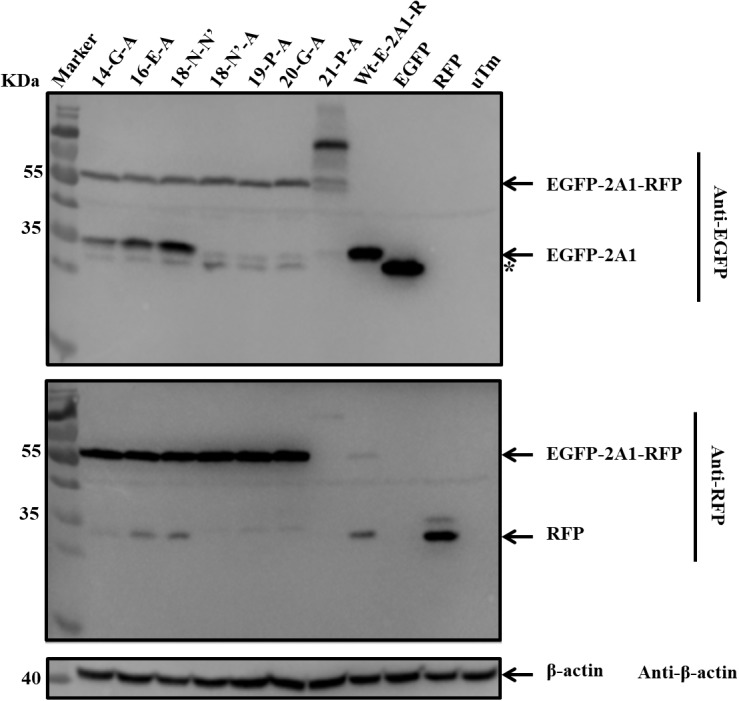
The influence of critical amino acids on ribosomal “skipping” function. The top half was immunoblotted with EGFP mAb. The bottom half was immunoblotted with RFP mAb. 14-G-A represents pcDNA3.1-E-20-R-G^14^-A. 16-E-A represents pcDNA3.1-E-20-R-E^16^-A. 18-N-N’ represents pcDNA3.1-E-20-R-N^18^-N’. 18-N’-A represents pcDNA3.1-E-20-R-N^18^-A. 19-P-A represents pcDNA3.1-E-20-R-P^19^-A. 20-G-A represents pcDNA3.1-E-20-R-G^20^-A. 21-P-A represents pcDNA3.1-E-20-R-P^21^-A. Asterisk refers unusual translational profiles. Two experimental materials were from the same batch of samples.

Based on the conservation of sequence alignment, we speculated that it was the motif “-G^14^xE^16^xN^18^P^19^G^20^P^21^-” mediating the ribosomal “skipping” function (in the motif, the number indicates the position of this amino acid in 2A1 when counting from its N-terminus; for convenience, the P of 2A2 is represented as P^21^). Therefore, we examined which amino acids were essential for this function in the short motif. We mutated all the amino acids to alanine in the motif and observed the influence on its function (Table 3). As can be seen (Figure 8), mutated alanine at positions 14, 16 weakened the ribosomal “skipping” function, but did not abolish it. A small amount of the divided proteins could still be detected. In contrast, mutations alanine at positions 18, 19, and 20 completely abolished the function. As a result, only large fusion proteins were detected and no small proteins were detected at all. At positions 21, weakened fusion and divided proteins were detected, but there is a larger band were detected, this may be due to mutations leading to protein multimer formation. From the above, we concluded that N^18^, P^19^, G^20^, and P^21^ actually play pivotal roles in the ribosomal “skipping” function, while other amino acids play important supporting roles. We also saw that the synonymous mutation of N^18^ had no effect on this function. It is suggestive that this position only affected by changes of amino acid, and is not affected by base changes.

**Table 3:** Alanine scanning mutation of the key amino acids.

site	14		16		18	19	20	B_1_
aa	G	x	E	x	N	P	G	P
mut_1_-aa	A		A		N’	A	A	A
mut_2_-aa					A			

## Discussion

To date, many studies have reported on 2A proteins in *Picornaviridae*. In many viruses, the 2A protein is involved in capsid assembly and virulence (Emerson et al., 2002), whereas the 2A peptide of FMDV is not (Gullberg et al., 2013). We think that 2A1 in DHAV-1, similar to the FMDV 2A, is also not involved in capsid formation. In this paper, we have shown that the DHAV-1 2A1 has a ribosomal “skipping” function. In other research, it has been reported that the D mutation of the classical FMDV “-DxExNPGP-” motif abolished this function (Donnelly et al., 2001a). However, our study showed that the “-GxExNPGP-” in DHAV-1 2A1 can still function successfully. Moreover, the mutation of the G residue to alanine just reduces the “skipping” level; it does not completely abolish the activity. From the alanine scanning mutagenesis of the key amino acids, we formed a different view of the research of FMDV. We think in fact that the key amino acids are N^18^P^19^G^20^P^21^. However, we can also see that the amino acids in this motif are all important for the ribosomal “skipping” function. Some mutations do not abolish the activity, but each mutation has a harmful influence on it. Therefore, we still regard the classical motif as the best match for this function. In recent study, the corresponding P^17^G^18^P^19^ in FMDV motif are reported essential for cotranslational “cleavage” (Kjaer and Belsham, 2018a). In addition, synonymous mutations of N^18^ have no influence on the activity. A study has already shown that the silent mutation of G/P in FMDV 2A has no influence on the motif function (Gao et al., 2014). However, a clear codon preference encoding the NPGP motif within FMDV was observed. The codons encoding P^17^ and P^18^ are not equivalent (Kjaer and Belsham, 2018b). The 2A of encephalomyocarditis virus also mediates a ribosomal “skipping” function. In addition, the virus also mediates a ribosomal frameshifting function; and the frameshift signal is located just downstream of the ribosomal “skipping” motif, but these two sequences do not affect the function of the other party (Napthine et al., 2017).

In addition, we constructed different lengths of 2A1 by extending or shortening its N-terminus. We found that the N-terminal length of the ribosomal “skipping” motif seriously affected the function. As the number of N-terminal amino acids increased, the level of ribosomal “skipping” improved. This characteristic is similar to the FMDV 2A protein (Donnelly et al., 1997). When VP1 + 2A1 was 30aa, the ribosome could “skip” completely. A decrease in the number of N-terminal amino acids in 2A1 reduced the level of ribosomal “skipping.” In addition, protein cleavage is not due to RNA cleavage or RNA transcription abnormalities, and the expressed protein level is also independent of RNA transcriptional level. This shows that although it is now recognized that the main motif for performing this function is “-GxExNPGP-,” our research is suggestive that the N-terminus of 2A1 also assists in the completion of this activity; because it cannot perform this function at all when 2A1 only has 10 amino acids at the C-terminal. It has been reported that FMDV 2A protein needs at least 13aa to carry out its function (Ryan and Drew, 1994). So how can one achieve an optimal combination of motif length and functional activity when the motif is applied to a practical application? The answer to this is a matter of great importance and challenge. In addition, if a different sequence is added between the FMDV 2A and the upstream reporter gene, it was found that different sequences have certain influences on the ribosomal “skipping” activity (Minskaia and Ryan, 2013). The research has proved that undivided fusion protein does not separate over time, which is a further indication that the motif function is mediated by translation but not proteolysis. Protein separation occurs during translation and external factors do not affect it.

Co-expression technology has worked in all eukaryotic systems tested and has been used in diverse areas, such as human cancer gene therapies, genetic engineering of human stem cells, co-expression of transcription factors in the induction of pluripotent stem cells, and the creation of transgenic plants and animals (Luke and Ryan, 2018). Not only is the 2A sequence smaller (54–174 bp) than IRES elements (∼600 bp), but also 2A co-expression of proteins is independent of the cell type. Caveats of the 2A system are: (i) 2A remains as a C-terminal extension of the upstream protein, and (ii) proline forms the N-terminus of the downstream protein. The presence of the 2A, however, can be used for detection and/or immuno-precipitation using antibodies to the 2A peptide.

## Author Contributions

XY conceived, designed and performed the experiments, analyzed the data and wrote the paper. QZ, MW, and AC conceived and designed the experiments. KP, DZ, ML, RJ, QY, YW, SC, XZ, SZ, YL, YY, and LZ interpreted the data. All authors read and approved the final manuscript for publication.

## Conflict of Interest Statement

The authors declare that the research was conducted in the absence of any commercial or financial relationships that could be construed as a potential conflict of interest.
