# Supplementary material for: DHAV-1 2A1 Peptide – A Newly Discovered Co-expression Tool That Mediates the Ribosomal “Skipping” Function
Source: Front Microbiol. 2018 Nov 15;9:2727. doi: 10.3389/fmicb.2018.02727 (PMC6249498; doi:10.3389/fmicb.2018.02727)
Supplement: Supplementary file 1 [file Data_Sheet_1.docx]

Supplementary Material

DHAV-1 2A1 peptide - A Newly Discovered Co-expression Tool that Mediates the Ribosomal ‘Skipping’ Function

Xiaoyao Yang^1,2,3+^, Qiurui Zeng^4+^, Mingshu Wang^1,2,3+^, Anchun Cheng^1,2,3*^, Kangcheng Pan^2^, Dekang Zhu^2,3^, Mafeng Liu^1,2,3^, Renyong Jia^1,2,3^, Qiao Yang^1,2,3^, Ying Wu^1,2,3^, Shun Chen^1,2,3^, Xinxin Zhao^1,2,3^, Shaqiu Zhang^1,2,3^, Yunya Liu^1,2,3^, Yanling Yu^1,2,3^, Ling Zhang^1,2,3^

*** Correspondence:** Anchun Cheng: chenganchun@vip.163.com

# Supplementary Figures and Tables


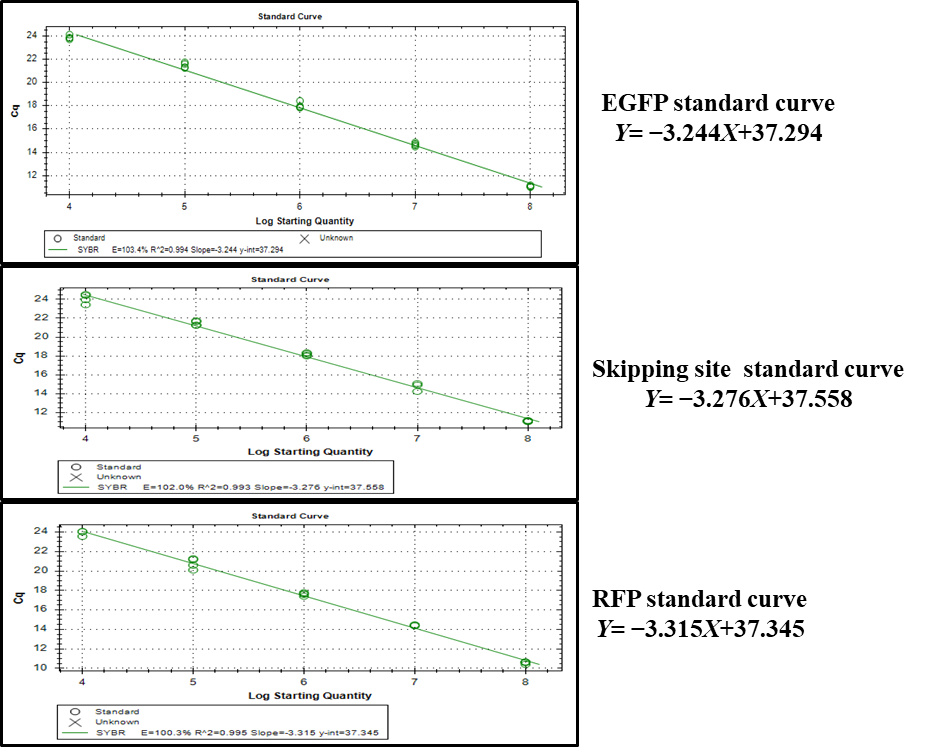


**Supplementary Figure 1.** The standard cure of EGFP, skipping site and RFP fragment respectively for qRT-PCR. Their detection range are all from 10^4^~10^8^ copies/μl
